# Activity of the yeast vacuolar TRP channel TRPY1 is inhibited by Ca^2+^–calmodulin binding

**DOI:** 10.1016/j.jbc.2021.101126

**Published:** 2021-08-28

**Authors:** Mahnaz Amini, Yiming Chang, Ulrich Wissenbach, Veit Flockerzi, Gabriel Schlenstedt, Andreas Beck

**Affiliations:** 1 Experimentelle und Klinische Pharmakologie und Toxikologie/PZMS, Universität des Saarlandes, Homburg, Deutschland; 2 Department of Medical Biochemistry and Molecular Biology/PZMS, Medical School, Saarland University, Homburg, Germany

**Keywords:** ion channel, TRPY1, Yvc1, vacuole, patch clamp, Ca^2+^ imaging, ^45^Ca^2+^, calcium channel, calmodulin, [Ca^2+^]_cyt_, cytosolic Ca^2+^ concentration, CaM, calmodulin, cDNA, complementary DNA, Cmd1, yeast calmodulin, ER, endoplasmic reticulum, GST, glutathione-*S*-transferase, HEK-293, human embryonic kidney 293, MBP, maltose-binding protein, TRP, transient receptor potential, TRPL, TRP like, TRPM, transient receptor potential melastatin, TRPV, transient receptor potential vanilloid, TRPY1, transient receptor potential yeast channel 1, YVC1, yeast vacuolar conductance 1

## Abstract

Transient receptor potential (TRP) cation channels, which are conserved across mammals, flies, fish, sea squirts, worms, and fungi, essentially contribute to cellular Ca^2+^ signaling. The activity of the unique TRP channel in yeast, TRP yeast channel 1 (TRPY1), relies on the vacuolar and cytoplasmic Ca^2+^ concentration. However, the mechanism(s) of Ca^2+^-dependent regulation of TRPY1 and possible contribution(s) of Ca^2+^-binding proteins are yet not well understood. Our results demonstrate a Ca^2+^-dependent binding of yeast calmodulin (CaM) to TRPY1. TRPY1 activity was increased in the *cmd1–6* yeast strain, carrying a non–Ca^2+^-binding CaM mutant, compared with the parent strain expressing wt CaM (Cmd1). Expression of Cmd1 in *cmd1–6* yeast rescued the wt phenotype. In addition, in human embryonic kidney 293 cells, hypertonic shock-induced TRPY1-dependent Ca^2+^ influx and Ca^2+^ release were increased by the CaM antagonist ophiobolin A. We found that coexpression of mammalian CaM impeded the activity of TRPY1 by reinforcing effects of endogenous CaM. Finally, inhibition of TRPY1 by Ca^2+^–CaM required the cytoplasmic amino acid stretch E_33_–Y_92_. In summary, our results show that TRPY1 is under inhibitory control of Ca^2+^–CaM and that mammalian CaM can replace yeast CaM for this inhibition. These findings add TRPY1 to the innumerable cellular proteins, which include a variety of ion channels, that use CaM as a constitutive or dissociable Ca^2+^-sensing subunit, and contribute to a better understanding of the modulatory mechanisms of Ca^2+^–CaM.

The transient receptor potential yeast (TRPY) channel 1 is a member of the superfamily of transient receptor potential (TRP) cation channels. Based on their amino acid sequence similarities, the TRP proteins fall into seven subfamilies, six of which are found in mammals: canonical (TRP canonical), vanilloid (TRP vanilloid [TRPV]), melastatin (TRP melastatin [TRPM]), mucolipin (TRP mucolipin), ankyrin (TRP ankyrin), and polycystin (TRP polycystin). The TRPN (NO-Mechano-Potential) has so far only been detected in worm, fly, and zebrafish and is proposed to be a mechanosensing channel. So far, no TRP channels have been identified in archaea or bacteria, and only few examples of TRP genes have been identified in nonland plants and fungi (1).

TRPY1 is the only TRP homolog in the yeast *Saccharomyces cerevisiae*. It is encoded by the gene *YVC1* (yeast vacuolar conductance 1) and forms ion channels in the yeast vacuolar membrane (2, 3, 4, 5). Recently, the Moissenkova–Bell group described the full-length structure of TRPY1 by cryo-EM with typical tetrameric TRP channel architecture, in which subunits are arranged in a four-fold symmetry around a central ion permeation path, as previously suggested (1, 6, 7) but with distinct structural folds for the cytosolic N and C termini (8).

TRPY1 is activated by osmotic stress (9, 10, 11), indole and related aromatic compounds (12), and Ca^2+^ acting from the cytosolic site (2, 13, 14). Reducing agents substantially lower the cytosolic Ca^2+^ concentration ([Ca^2+^]_cyt_) required for TRPY1 activation from the cytosol (2, 14) by targeting cysteine residue 624 (4) to EC_50_ values of 391 to 498 μM (13). The resting level of cytoplasmic Ca^2+^ in yeast is about 200 to 400 nM (15, 16), and it has been suggested (17) that hyperosmotic shock represents the initial physiological stimulus in intact yeast, which elevates [Ca^2+^]_cyt_ by Ca^2+^ release *via* TRPY1, and thereby reinforces TRPY1 activity (13). A negative charge cluster D^573^DDD^576^ within the cytoplasmic C terminus has been postulated as binding site on TRPY1 for Ca^2+^, although a deletion TRPY1 mutant, lacking this negative charge cluster including adjacent amino acid residues (amino acids 570–580) retains a residual Ca^2+^ dependence (17). Apparently, additional Ca^2+^-dependent mechanisms are required to restrain TRPY1 activation.

Bertl and Slayman (18) proposed a Ca^2+^–calmodulin (CaM)-dependent mechanism contributing to TRPY1 regulation. CaM is a ubiquitous and highly conserved Ca^2+^-binding protein conveying changes of cytoplasmic Ca^2+^ concentrations to numerous target proteins including ion channels. *In vitro*, single and multiple binding sites for Ca^2+^–CaM have been identified in protein fragments of the mammalian TRPs (19) and of the founding members of the TRP channel family, *Drosophila* TRP and TRP like (TRPL) (20, 21, 22, 23, 24). Ca^2+^–CaM binding has been linked to TRPV1 desensitization (25), TRP ankyrin channel 1 potentiation and inactivation (26), TRPM4 sensitization (27), and TRPV5 and TRPV6 inhibition (28, 29, 30). Different from *in vitro* binding studies and published CaM structures bound to ion channel fragments *in vitro*, according to recent cryo-EM structures, TRPV5 and TRPV6 inhibition required one CaM molecule per TRPV6 (30) or TRPV5 tetramer (31, 32). Up to six surface regions of TRPV6 or TRPV5 interact with the single CaM, which contacts all four subunits of the tetramer.

Mammalian CaM comprises two globular domains, the N and C lobes. Each lobe contains two EF-hand–type Ca^2+^-binding motifs (33, 34). The N and C lobes are connected by a central flexible linker that allows CaM and Ca^2+^–CaM to differently interact with target proteins including TRP channels. Sequence identity of the well-conserved mammalian CaMs and yeast CaM is 60% (34). Yeast CaM (Cmd1) has four potential high-affinity Ca^2+^-binding loops, but only the first three loops have all the residues required to bind Ca^2+^ (35). The yeast strain relying only on the non–Ca^2+^-binding CaM mutant Cmd1–6 is viable, whereas disruption of the wt CaM gene (*cmd1*) is lethal (35).

Here, we characterize TRPY1 as a Ca^2+^–CaM-binding protein. Ca^2+^ imaging and vacuolar patch clamp recordings in wt and *cmd1–6* yeast reveal TRPY1 channel inhibition depending on Ca^2+^–CaM. Similarly, activity of TRPY1 expressed in human embryonic kidney 293 (HEK-293) cells is inhibited by overexpressed mammalian CaM but not by the mammalian non–Ca^2+^-binding CaM mutant CaM(D_EF1,2,3,4_A). Channel inhibition was abolished by deleting a stretch of 60 amino acids within the N terminus of TRPY1, apparently representing major sites of CaM's contact with TRPY1.

## Results

### TRPY1 interacts with Ca^2+^–CaM *in vitro*

**Figure 1:**
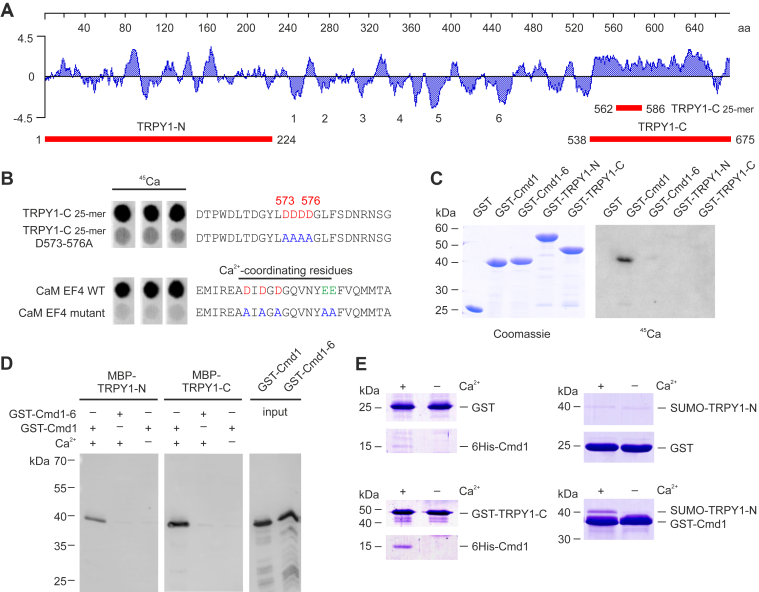
**TRPY1 interacts with Ca**^**2+**^**–CaM *in vitro*.***A*, hydrophilicity plot of TRPY1 with predicted transmembrane domains 1 to 6. Recombinant protein fragments corresponding to the cytosolic N terminus (TRPY1-N), C terminus (TRPY1-C), and a 25-amino acid (amino acids; 25-mer) peptide (TRPY1-C 25-mer) are shown in *red*. *B*, autoradiographs of ^45^Ca^2+^ binding by 25-mer peptides of wt and mutant TRPY1 (TRPY1-C 25-mer; *upper panel*) and wt and mutant CaM EF-hand 4 (CaM EF4; *lower panel*). Aspartate (D, *red*) and glutamate (E) residues (*green*) replaced by alanine (A, *blue*) are indicated. *C*, Coomassie stain (*left*) and autoradiograph of ^45^Ca^2+^ binding (*right*) of the recombinant proteins, fused to glutathione-*S*-transferase (GST). Yeast CaM (Cmd1) served as positive and the non–Ca^2+^-binding yeast CaM mutant (Cmd1–6) as negative control. *D*, binding of GST-fused Cmd1 or Cmd1–6 to maltose-binding protein (MBP)–fused TRPY1-N and TRPY1-C protein fragments in the absence (5 mM EGTA) or presence of 2 mM Ca^2+^, visualized by anti-GST antibody. Input controls are shown in the two *right lanes*. *E*, Coomassie staining of 6His-Cmd1 binding to GST or the GST-TRPY1-C protein fragment (*left*) and 6His-Sumo-TRPY1-N fusion protein binding to GST or GST-Cmd1 (*right*) in the absence and presence of Ca^2+^. CaM, calmodulin; TRPY1, transient receptor potential yeast channel 1.

The yeast vacuolar TRP channel (TRPY1, Yvc1; hydrophilicity plot) (Fig. 1*A*) is activated by cytoplasmic Ca^2+^ (2, 5, 13, 14). Ca^2+^ was suggested to interact with the negative charge cluster D^573^DDD^576^ within the C terminus of TRPY1 assumed to be exposed to the cytoplasm (17). A 25-amino acid peptide covering this negative charge cluster and adjacent amino acid residues, spotted to a cellulose membrane, significantly bound ^45^Ca^2+^ (Fig. 1*B*, *top*). The Ca^2+^ binding was significantly reduced when D^573^DDD^576^ were replaced by alanine residues. As positive and negative controls, 25-mer peptides of the fourth Ca^2+^-binding loop of mammalian CaM and its D^7,9,11^A, E^17,18^A mutant version were treated in the same way (Fig. 1*B*, *bottom*). Next, we fused the TRPY1 N and C terminus (224 and 137 amino acids) to glutathione-*S*-transferase (GST) and tested the fusion proteins TRPY1-N and TRPY1-C (Fig. 1*C*) for direct Ca^2+^ binding. GST or GST-fused yeast CaM (GST-Cmd1) and its non–Ca^2+^-binding mutant version Cmd1–6 (35) served as controls. In the mutant Cmd1–6, the aspartate and glutamate residues at the first and 12th position of the three Ca^2+^-binding loops were replaced by alanine residues. While all proteins were detected by Coomassie blue (Fig. 1*C*, *left*), only GST-Cmd1 significantly bound ^45^Ca^2+^ (Fig. 1*C*, *right*). Apparently, the negative charge cluster D^573^DDD^576^ involved in Ca^2+^ binding of the 25-mer peptide is buried in protein domains present in the C terminus of the 137 amino acids (GST-TRPY1-C (538–675)) and thus could not be accessed by Ca^2+^.

We therefore analyzed for a potential interaction of Ca^2+^–CaM and TRPY1. Pull-down assays were performed using recombinant TRPY1-N and TRPY1-C fused to maltose-binding protein (MBP) and GST-fused Cmd1 and Cmd1–6 proteins. Purified MBP-TRPY1-N and MBP-TRPY1-C were immobilized to amylose resin and incubated with GST-Cmd1 or GST-Cmd1–6 in the absence and presence of 2 mM Ca^2+^. Cmd1 but not Cmd1–6 significantly bound to both protein fragments of TRPY1 but only in the presence of Ca^2+^ (Fig. 1*D*). To confirm the latter interactions, we produced TRPY1-N and TRPY1-C with additional tags. GST-tagged TRPY1-C but not GST alone binds 6His-Cmd1 in the presence of Ca^2+^ (Fig. 1*E*, *left*). Similarly, the SUMO-TRPY1-N fusion protein strongly bound GST-Cmd1, but not GST alone, again in a Ca^2+^-dependent manner (Fig. 1*E*, *right*). The data suggest at least one interaction site of CaM at both the N and C terminus of TRPY1. This binding requires Ca^2+^ and a CaM molecule with intact Ca^2+^-binding sites.

### Ca^2+^–CaM inhibits the activity of TRPY1 in yeast

**Figure 2:**
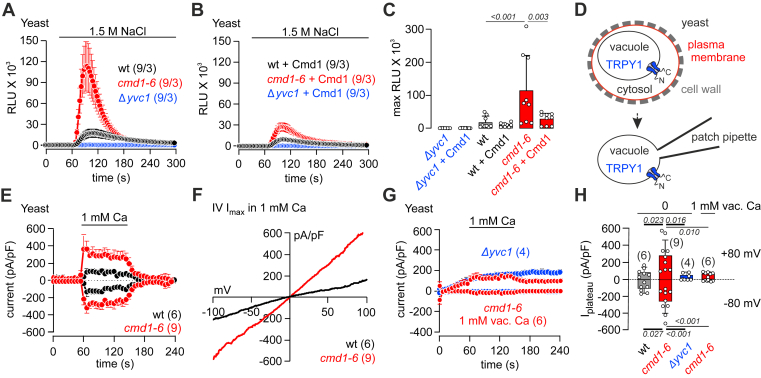
**Ca**^**2+**^**–CaM inhibits the activity of TRPY1 in yeast.***A* and *B*, changes of cytosolic Ca^2+^ challenged by 1.5 M NaCl (hyperosmotic shock, indicated by *black line*) monitored in wt, CaM-mutant *cmd1–6* and yvc1-deficient (*Δyvc1*) yeast strains in the absence (*A*) and presence (*B*) of transformed wt yeast CaM (Cmd1) as relative luminescence units (RLUs). *Numbers in parentheses* (n/x) indicate the number of experiments n from x independent yeast preparations. *C*, summary of the changes of cytosolic Ca^2+^ in *A* and *B*. *D*, zymolyase-dependent release of yeast vacuoles for patch clamp experiments. *E* and *G*, inward and outward currents at −80 and 80 mV, extracted from 200 ms voltage ramps (0.5 Hz) spanning from 150 to −150 mV, V_h_ = 0 mV, plotted *versus* time, measured in vacuoles from wt (*black*; *E*), *cmd1–6* (*red*; *E* and *G*), and *Δyvc1* (*blue*; *G*) yeast strains in the absence (*E* and *Gblue*) and presence (*red*; *G*) of 1 mM vacuolar Ca^2+^, which corresponds to the [Ca^2+^] in the patch pipette. The *bar* indicates application of 1 mM Ca^2+^ from the cytosolic side. *F*, corresponding current–voltage relationships (IVs) of the maximal currents (*I*_max_) in *E*. *H*, amplitudes of the net 1 mM Ca^2+^-mediated plateau currents at −80 and 80 mV from experiments in *E* and *G*, with current amplitudes right before application of 1 mM Ca^2+^ subtracted. The *numbers in brackets* (*E*–*H*) indicate the number of measured vacuoles. Data represent means (*F*) and means ± SEM (*A*, *B*, *E*, and *G*) or means ± SD (*C* and *H*) with *p* values in *C* and *H* calculated by one-way ANOVA (ANOVA values: *C* and *F* = 8.320, *p* < 0.0001; *H* for +80 mV, *F* = 6.761, *p* = 0.0023; *H* for −80 mV, *F* = 12.41, *p* < 0.0001) with Bonferroni's multiple comparison test. CaM, calmodulin; Cmd1, yeast calmodulin; TRPY1, transient receptor potential yeast channel 1.

To study the effect of CaM on TRPY1 activity in intact yeast cells, we expressed the luminescent Ca^2+^ reporter aequorin in wt yeast, in yeast where endogenous *cmd1* was replaced by *cmd1–6* and in TRPY1 knockout yeast (*Δyvc1*). TRPY1 activity was challenged by increasing the osmolarity in the bath solution (3, 5, 13, 14). Application of 1.5 M NaCl (hyperosmotic shock) induced a cytoplasmic Ca^2+^ signal in wt yeast, which was not detectable in *Δyvc1* cells but massively increased in *cmd1–6* cells (Fig. 2, *A* and *C*). Overexpression of *cmd1* slightly reduced the hyperosmotic shock–induced Ca^2+^ signal in wt cells but significantly diminished the Ca^2+^ signal in the *cmd1–6* cells (Fig. 2, *A*–*C*). Thus, the ability of CaM to bind Ca^2+^ is required to inhibit TRPY1 activity.

TRPY1 is located in the yeast vacuolar membrane, and next, we recorded TRPY1 currents from wt and *cmd1–6* yeast vacuoles by patch clamp experiments (Fig. 2*D*). For intracellular organelles, like the vacuole, membrane potentials refer to the cytosolic side and inward currents, for example, at −80 mV, represent movement of positive charges out of the vacuole into the cytosol (36). Application of 1 mM Ca^2+^ or 1 mM Ba^2+^ from the cytosolic side induced inward and outward currents, which were significantly increased in *cmd1–6* compared with wt vacuoles (Fig. 2, *E*, *F*, and *H* and Fig. S1, *A*–*C*). Vacuoles, isolated from *Δyvc1* cells, did not reveal any inward current upon application of 1 mM cytosolic Ca^2+^ (Fig. 2*G*, *blue trace*) or Ba^2+^ (Fig. S1*D*). The outward current (Fig. 2*G* and Fig. S1*D*) represents a vacuolar Cl^−^ conductance (13), which is also present when TRPY1 activity is abolished by 1 mM vacuolar Ca^2+^ (Fig. 2*G*, *red trace*, and Fig. 2*H*). The TRPY1-independent Cl^−^ current is slightly increased by cytosolic application of Ca^2+^ (Fig. 2*G*) and even more by Ba^2+^ (Fig. S1*D*).

### CaM modulates the activity of TRPY1 expressed in HEK-293 cells

**Figure 3:**
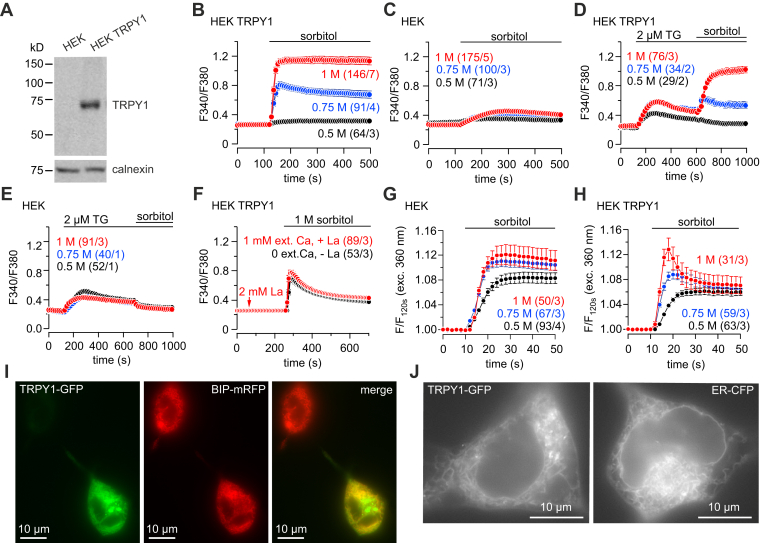
**Hyperosmotic shock by application of 1 M sorbitol activates Ca**^**2+**^**influx and Ca**^**2+**^**release in TRPY1-expressing HEK-293 cells.***A*, Western blot of protein lysates from HEK-293 cells transfected with the TRPY1 complementary DNA without (*left lane*) and after induction (*right lane*) with ponasterone A. The TRPY1 protein was detected by the monoclonal anti-TRPY1 antibody. The filter was stripped and incubated with antibody for calnexin (*lower panel*) as loading control. *B*–*H*, HEK-293 cells transfected with *YVC1-IRES-GFP* (*B*, *D*, *F*, and *H*) or *IRES-GFP* only as controls (*C*, *E*, and *G*) were challenged by application of sorbitol or thapsigargin (TG) to the bath solution at the concentrations indicated, and changes of the cytosolic [Ca^2+^] were measured as Fura-2 fluorescence ratio (F340/F380) in the presence of 1 mM extracellular Ca^2+^ (*B*–*F*) or the absence of extracellular Ca^2+^ (*F*, *black trace*). The *arrow* in *E* indicates application of 2 mM La^3+^ (*red trace*) in the presence of extracellular Ca^2+^. *G* and *H*, volume changes were measured as Fura-2 fluorescence after excitation at the isosbestic wavelength 360 nm (F/F_120s_). *Numbers in brackets* (n/x) indicate the total number of measured cells n from x separately transfected dishes. Data represent means ± SEM. *I* and *J*, fluorescence images of HEK-293 cells transiently transfected with complementary DNAs of TRPY1 3′ extended by the complementary DNA (cDNA) of GFP to yield TRPY1-GFP fusion proteins (*I* and *J*, *left*), the cDNA of the ER-resident chaperon BiP, fused to mRFP cDNA (BiP-mRFP; *I*), and cDNA of the ER marker ER-CFP (*J*, *right*). ER, endoplasmic reticulum; HEK-293, human embryonic kidney 293 cells; TRPY1, transient receptor potential yeast channel 1.

TRPY1 retains its channel properties when expressed in HEK-293 cells (13), and we established a Fura-2-based Ca^2+^ imaging protocol to monitor TRPY1 activity in the absence and presence of increasing osmolarity of the Ca^2+^-containing bath solution by application of sorbitol. TRPY1 complementary DNA (cDNA) expression was induced in transfected HEK-293 cells by ponasterone A. The TRPY1 protein was detectable in induced but not in noninduced transfected HEK-293 cells (Fig. 3*A*). For Ca^2+^ imaging, HEK-293 cells were identified by their GFP expression, that is, *green fluorescence*. Application of sorbitol dose dependently caused a cytoplasmic increase of Ca^2+^ in TRPY1-expressing cells (Fig. 3*B*) but not in control cells only expressing GFP (Fig. 3*C*). After depleting intracellular Ca^2+^ stores by thapsigargin, this cytosolic Ca^2+^ increase in the presence of sorbitol remained (Fig. 3*D*), whereas sorbitol had no effect on HEK-293 control cells (Fig. 3*E*). Thus, TRPY1, present in the plasma membrane, is responsible for the hyperosmotic shock–induced Ca^2+^ influx. Application of sorbitol in the absence of extracellular Ca^2+^ still elicits an increase of cytoplasmic Ca^2+^, which equals the sorbitol-induced Ca^2+^ increase in the presence of extracellular Ca^2+^ when the plasma membrane TRPY1 is blocked by La^3+^, a Ca^2+^ flux inhibitor (Fig. 3*F*). Such Ca^2+^ signals are not seen in HEK-293 control cells upon stimulation with 1 M sorbitol even in the presence of extracellular Ca^2+^ (Fig. 3*C*). Apparently, TRPY1, transiently expressed in HEK-293 cells, is also present in the membrane of the endoplasmic reticulum (ER) and, in addition to Ca^2+^ influx (see aforementioned), mediates a hyperosmotic shock–induced Ca^2+^ release.

To show the localization of TRPY1, we 3′ fused GFP cDNA to the cDNA of TRPY1. HEK-293 cells were transfected with the new cDNA to yield the TRPY1-C-GFP fusion protein (TRPY1-GFP). The cDNA of BiP, a chaperone located in the lumen of the ER, fused to mRFP (BiP-mRFP), was coexpressed as a marker for the ER (Fig. 3*I*). Independently, another marker for the ER, ER-CFP (pECFP-ER; Clontech), was expressed and compared with the expression of TRPY1-GFP (Fig. 3*J*). TRPY1-GFP and BiP-mRFP are colocalized (Fig. 3*I*, *yellow cell*) and TRPY1-GFP and ER-CFP exhibit the same cellular fluorescence pattern (Fig. 3*J*). The fluorescence images show that TRPY1 is present in ER membranes and functionally contributes to hyperosmotic shock–mediated Ca^2+^ release from intracellular Ca^2+^ stores (Fig. 3*F*). Infusion of 1 μM Ca^2+^ *via* the patch pipette revealed whole-cell currents in TRPY1-GFP–expressing cells but not in HEK-293 control cells (Fig. S2, *A* and *B*), showing that the TRPY1-GFP fusion protein forms a functional channel in the plasma membrane, as unfused TRPY1 does.

The cells shrink in the hyperosmotic bath solution, and we analyzed volume changes by exciting Fura-2 fluorescence at the isosbestic wavelength 360 nm and monitored the emitted light using the same filter system as for Ca^2+^ measurements (Fig. 3, *G* and *H*). Hypertonic shock mediated a fluorescence increase, that is, volume reduction, of HEK-293 control cells in a dose-dependent manner (Fig. 3*G*), which was more transient and significantly reduced after an initial peak in TRPY1-expressing cells (Fig. 3*H*). The initial volume reduction activates TRPY1, and cation influx into the cell occurs, counteracting the hyperosmotic shock–induced volume reduction only in TRPY1-expressing cells.

These results show that in HEK-293 cells, TRPY1 present in the plasma membrane and in the membranes of intracellular Ca^2+^ stores mediate the hyperosmotic shock–induced increase of cytoplasmic Ca^2+^ and that TRPY1 at both places may contribute to a TRPY1 function in osmoregulation.

**Figure 4:**
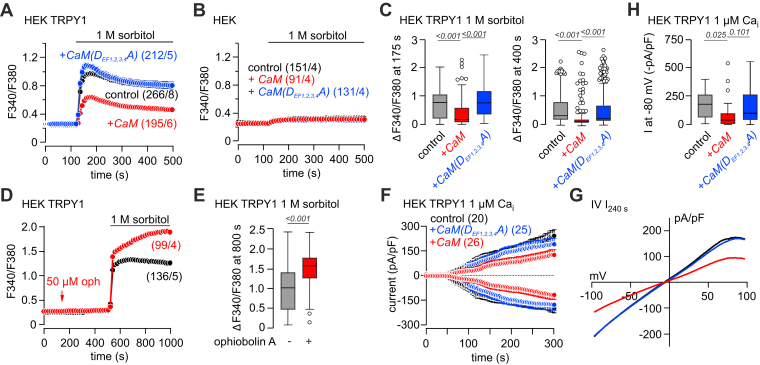
**Mammalian calmodulin modulates the activity of TRPY1 expressed in HEK-293 cells.** Cytosolic [Ca^2+^] changes, measured as Fura-2 fluorescence ratio (F_340_/F_380_), in HEK-293 cells transfected with *YVC1-IRES-GFP* (*A* and *D*) or *IRES-GFP* complementary DNA (*B*) challenged by 1 M sorbitol (*black bar*). In *A* and *B*, cells were cotransfected with mammalian calmodulin (CaM; *red*), the non–Ca^2+^-binding calmodulin mutant (CaM(D_EF1,2,3,4_A); *blue*), or empty vector as control (*black*). *Arrow* in *D* indicates application of 50 μM ophiobolin A (oph, *red trace*). *F*, inward and outward currents at −80 and 80 mV, extracted from 400 ms voltage ramps (0.5 Hz) spanning from −100 to 100 mV, V_h_ = 0 mV, plotted *versus* time, measured in HEK-293 cells transfected with *YVC1-IRES-GFP* complementary DNA challenged by 1 μM intracellular Ca^2+^ (Ca_i_) infused *via* the patch pipette. Cells were cotransfected with mammalian calmodulin (CaM; *red*), the non–Ca^2+^-binding calmodulin mutant (CaM(D_EF1,2,3,4_A); *blue*) or empty vector as control (*black*). *G*, corresponding current–voltage relationships (IVs) of the currents at 240 s (I_240 s_) in *F*. The *numbers in brackets* (n/x) in *A*, *B*, and *D* indicate the total number of measured cells n from x separately transfected dishes. The *numbers in brackets* in *F* indicate the number of measured cells. *C*, *E*, and *H*, summary of the [Ca^2+^] increase at 175 s (*left*) and 400 s (*right*) in *A* (*C*), at 800 s in *D* (*E*), and the current amplitude at −80 mV extracted at 240 s in *F* (*H*). Data in *A*, *B*, *D*, and *F* represent means ± SEM. Data in *C*, *E*, and *H* are presented as Tukey's box, and whiskers with the *boxes* extend from the 25th to the 75th percentile (interquartile range [IQR]), and the *line* inside the *box* shows the median. Whiskers are extended to the most extreme data point that is no more than 1.5× IQR from the edge of the box, and outliers beyond the whiskers are depicted as *dots*. The indicated *p* values were calculated by Kruskal–Wallis test (*C*, *left*, *p* < 0.0001; *C*, *right*, *p* < 0.0001; *H*, *p* = 0.0183) with Dunn's multiple comparison test. HEK-293, human embryonic kidney 293 cells; TRPY1, transient receptor potential yeast channel 1.

Next, HEK-293 cells were cotransfected with the TRPY1 cDNA and the cDNAs of wt mammalian CaM or the mammalian non–Ca^2+^-binding CaM(D_EF1,2,3,4_A) mutant, carrying a D-to-A mutation in the first position of all four EF hands (37). The hyperosmotic shock–induced Ca^2+^ signal was significantly reduced in cells coexpressing TRPY1 and CaM compared with cells expressing TRPY1 alone or cells coexpressing TRPY1 and the CaM(D_EF1,2,3,4_A) mutant (Fig. 4, *A* and *C*). Neither CaM nor CaM(D_EF1,2,3,4_A) expression revealed any effect on the cytosolic Ca^2+^ upon application of 1 M sorbitol in HEK-293 control cells (Fig. 4*B*). Inhibition of TRPY1 expressed in HEK-293 cells by endogenous CaM depends, like in yeast, on Ca^2+^ binding, and is prevented by ophiobolin A, a CaM inhibitor (Fig. 4, *D* and *E*). In addition, TRPY1 whole-cell currents in HEK-293 cells, activated by 1 μM Ca^2+^ in the patch pipette, were significantly reduced after coexpression of CaM but not the CaM(D_EF1,2,3,4_A) mutant (Fig. 4, *F*–*H*). We also recorded TRPY1 currents induced by hyperosmotic shock (500 mM sorbitol) after overexpression of the CaM cDNA (Fig. S3, *H*–*K*) or activated by 1 μM intracellular Ca^2+^ and hyperosmotic shock (500 mM sorbitol) after cytosolic infusion of 10 μM recombinant mammalian CaM *via* the patch pipette (Fig. S3, *A*–*G*). In all protocols, cytosolic CaM decreased the TRPY1 current. In contrast, coexpression of the non–Ca^2+^-binding CaM mutant (CaM(D_EF1,2,3,4_)) had little effect on TRPY1 currents. The hyperosmotic shock–induced currents were not seen in nontransfected HEK-293 cells (Fig. S3, *L* and *M*) and were virtually abolished in TRPY1-expressing cells in the absence of intracellular Ca^2+^ (10 mM BAPTA, 0Ca_i_; Fig. S3, *N* and *O*).

The data also show that mammalian CaM can functionally replace yeast CaM in inhibiting the activity of TRPY1. Such a functional replacement of yeast CaM by its mammalian homolog has been shown for several physiological functions in yeast (15, 34, 38, 39).

### Cmd1-binding sites of the TRPY1 N terminus required for current inhibition

*In vitro*, Ca^2+^–Cmd1 is bound by both the N and C terminus of TRPY1 (Fig. 1), and according to online databases (40, 41), multiple and partially overlapping binding sites for mammalian CaM within the N and C terminus of the TRPY1 protein are predicted. In order to identify the binding sites for yeast CaM (Cmd1), we performed a peptide scan. Peptides corresponding to the N and C terminus of TRPY1, with a length of 20 amino acids each, were synthesized on a cellulose membrane, and the amino acid sequences were shifted by five amino acids from one spot to the other. After incubation of the cellulose membrane with ^14^C-labeled Cmd1 and exposure, we did not find clearly defined single sites but rather several binding domains. According to the relative intensity visualized on the autoradiograph (Fig. S4), two domains within the N terminus, residues 68 to 92, and less intense residues 28 to 52, were most prominent, compared with domains within the C terminus (residues 527–546 and residues 607–631).

**Figure 5:**
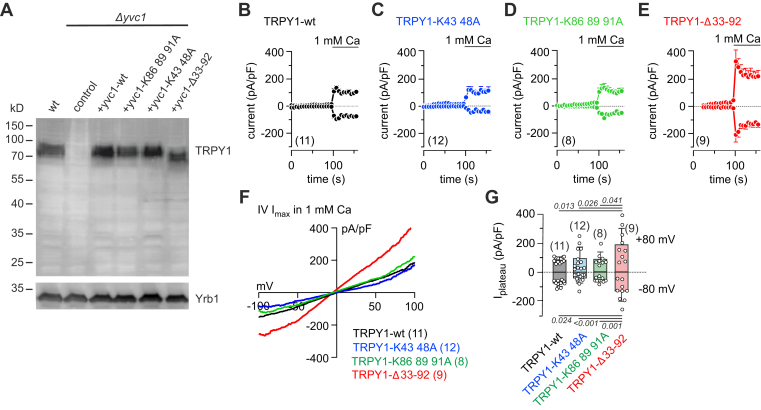
**Cmd1 binding sites of the TRPY1 N terminus required for current inhibition.***A*, Western blot of protein lysates from wt yeast strain (wt), TRPY1 knockout (*Δyvc1*) yeast strain (control), and *Δyvc1* transformed with single copy plasmids of wt YVC1, YVC1-K86 89 91A, YVC1-K43 48A, and YVC1-Δ33 to 92 incubated in the presence of the monoclonal anti-TRPY1 antibody. The filter was stripped and incubated with antibody for Yrb1 (*lower panel*) as loading control. *B*–*F*, inward and outward currents at −80 and 80 mV, extracted from 200 ms voltage ramps (0.5 Hz) spanning from 150 to −150 mV, V_h_ = 0 mV, plotted *versus* time (*B*–*E*) and corresponding IVs of the maximal currents (*I*_max_, *F*), recorded from vacuoles isolated from *Δyvc1* yeast strain transformed with single copy plasmids of wt TRPY1 (*B*), YVC1-K43 48A (*C*), YVC1-K86 89 91A (*D*), and YVC1-Δ33 to 92 (*E*). The bar indicates cytosolic application of 1 mM Ca^2+^. *G*, amplitudes of the net plateau currents upon activation by 1 mM Ca^2+^ at −80 and 80 mV from experiments in *B*–*E*, with current amplitudes right before application of 1 mM Ca^2+^ subtracted. The *numbers in brackets* indicate the number of measured vacuoles. Data represent means (*F*) and means ± SEM (*B*–*E*) or means ± SD (*G*) with *p* values in *G* calculated by one-way ANOVA (ANOVA values: +80 mV, *F* = 4.638, *p* = 0.0077; −80 mV, *F* = 9.80, *p* < 0.0001) with Bonferroni's multiple comparison test. Cmd1, yeast calmodulin; TRPY1, transient receptor potential yeast channel 1.

Because most confirmed CaM-binding sites have a net positive charge, lysine residues of the major two domains at positions 43 and 48 as well as 86, 89, and 91 within the N terminus were replaced by alanine residues, and, in addition, both domains were deleted, generating TRPY1-K43 48A, TRPY1-K86 88 91A, and TRPY1-Δ33 to 92 cDNAs. The wt and the mutant cDNAs expressed in TRPY1 knockout yeast (*Δyvc1*) yielded comparable amounts of protein (Fig. 5*A*). Next, we recorded TRPY1 currents from yeast vacuoles by patch clamp experiments. Application of 1 mM Ca^2+^ from the cytosolic side induced inward and outward currents in vacuoles expressing wt TRPY1 (Fig. 5*B*). The current amplitudes of TRPY1-K43 48A (Fig. 5*C*) and TRPY1-K86 89 91A (Fig. 5*D*) were not distinguishable to the current amplitudes obtained from the wt channel (Fig. 5, *B*–*D*, *F*, and *G*), whereas deletion of residues 33 to 92 (TRPY-Δ33–92) including both domains increased current amplitude almost twofold (Fig. 5, *E*–*G*). As shown in Figure 2, *E*, *F*, and *H*, replacement of wt Cmd1 by cmd1–6 had a similar effect on wt TRPY1 currents.

## Discussion

The present study identifies TRPY1 as a Ca^2+^–CaM-binding protein. *In vitro*, purified recombinant TRPY1 protein fragments bound yeast CaM but not the non–Ca^2+^-binding yeast CaM mutant cmd1–6 in pull-down assays. *In vivo*, patch clamp recordings from yeast vacuoles and HEK-293 cells expressing TRPY1 and Ca^2+^ imaging experiments in yeast and HEK-293 cells demonstrate that Ca^2+^–CaM inhibits TRPY1 activity and that a 60 amino acid domain within the N terminus of TRPY1, exposed to the cytoplasm, is required for this inhibition.

CaM is a universal Ca^2+^ sensor, which translates changes of the [Ca^2+^]_cyt_ to target proteins including ion channels and thereby regulates target protein function. Based on the results of pull-down experiments with recombinant and purified fragments of TRPY1 and yeast CaM (Fig. 1, *D* and *E*), we performed a peptide scan in order to identify Ca^2+^–CaM binding sites within the N and C terminus of TRPY1. We did not find clearly defined single sites but rather several CaM-binding domains (Fig. S4). This is not an exceptional finding because CaM dynamically binds to its targets, which may not be represented by a single linear binding motif as illustrated by recent structures of the small-conductance Ca^2+^-activated K^+^ (SK) channel (42) or TRPV6 and TRPV5 channels in complexes with Ca^2+^–CaM (30, 31, 32). TRPV5 and TRPV6 channels undergo Ca^2+^-induced inactivation, proposed to contain a fast Ca^2+^-dependent and a slow Ca^2+^–CaM-dependent component. More than five Ca^2+^–CaM binding sites have been identified in TRPV5 and TRPV6 proteins *in vitro* (28, 29, 30, 43, 44, 45). Synthesized as peptides or recombinant channel protein fragments, most of these different sites bound at least one CaM molecule *in vitro*, whereas in the TRPV5–CaM and TRPV6–CaM channel structures (30, 31, 32), one channel tetramer binds one single CaM molecule. It contacts a tryptophan residue adjacent to the channels' lower gate and various hydrophobic patches on the N terminus and on the C terminus of single subunits. In the human SK4 (KCNN4) channel–CaM complex, four CaM molecules bind to one channel tetramer with each single CaM molecule communicating with three channel subunits (42). Based on these structure data, single subunits of homotetrameric channels may assume different functions in terms of CaM binding.

Neither the tryptophan nor the sequence motifs of the hydrophobic patches are conserved in TRPY1, which shares less than 16% sequence identity with TRPV6 or TRPV5. We therefore replaced the lysine residues 43 and 48 (TRPY1-K43 48A) and the lysine residues 86, 89, and 91 (TRPY1-K86 89 91A) by alanine residues within those domains of TRPY1, which were most prominent in the peptide scan (domains E_28_ to I_52_ and S_68_ to K_102_, Fig. S4). Expressed in the TRPY1 knockout strain, the vacuolar TRPY1 currents were not distinguishable from wt TRPY1 (Fig. 5, *A*–*D*). However, a TRPY1 deletion mutant lacking amino acid domain R_33_ to Y_92_, covering most of the two domains identified by the peptide scan including the lysine residues, rescued the vacuolar current phenotype obtained with the non–Ca^2+^-binding CaM–expressing yeast strain (cmd1–6).

For vacuolar current recordings, we activated TRPY1 by applying Ca^2+^ to the bath solution. We have previously shown that Ca^2+^ applied from the cytosolic site activates vacuolar TRPY1 outward and inward currents at EC_50_ values of 391 and 498 μM, but the mechanism of Ca^2+^-mediated activation is yet not fully understood (2, 5, 10, 13, 14, 46, 47). As shown in Figure 1*B*, *in vitro*, Ca^2+^ binds to a linear 25-mer peptide containing the negative charge cluster D^573^DDD^576^ and adjacent amino acid residues from the cytosolic C terminus of TRPY1, suggested to be crucial for its cytosolic Ca^2+^-mediated activation (17). Four additional aspartate residues are located in close vicinity of the cluster, which might cause the residual Ca^2+^ binding to the peptide containing the A^573^AAA^576^ mutation (Fig. 1*B*). We did not observe any Ca^2+^ binding to the complete recombinant TRPY1 C terminus K_538_ to E_675_, where the accessibility of the cluster for Ca^2+^ might be prevented by structural restraints (Fig. 1*C*). Maintained TRPY1 channel activity was shown after replacing D^573^DDD^576^ by asparagine residues (4) and a residual Ca^2+^ dependence of the deletion mutant TRPY1Δ^570^G-S^580^ (17), whereas TRPY1Δ^570^G-L^600^ failed to respond to Ca^2+^ (12).

In addition to TRPY1, TRPV5, and TRPV6, a variety of additional TRP channel proteins contain CaM-binding sites. The founding members of the TRP channel superfamily, the fly photoreceptor TRPs, TRP and TRPL, were the first TRPs characterized as Ca^2+^–CaM-binding proteins. The photoreceptor TRP protein has at least two Ca^2+^–CaM binding sites in the C terminus. *In vitro*, peptides or recombinant fragments carrying these binding sites bound CaM only in the presence of Ca^2+^ (20, 22, 48). The second fly photoreceptor TRP channel, TRPL, was initially identified in a screen for CaM-binding proteins (49). Two CaM-binding sites were identified in TRPL fragments of its C terminus (23, 24). Both fly photoreceptor TRP channels are positively and negatively regulated by Ca^2+^ (50, 51), but whether CaM is involved in this regulation is not known and might depend on the heterologous expression systems, insect Sf9 cells, or mammalian CHO cells (21, 23, 52, 53).

We show here that TRPY1 in the yeast *S. cerevisiae* is under inhibitory control of Ca^2+^–CaM. This inhibition is maintained when TRPY1 is expressed in mammalian HEK-293 cells and when yeast CaM is replaced by mammalian CaM. In intact yeast, Ca^2+^–CaM might restrain excessive TRPY1 responses to Ca^2+^-induced Ca^2+^ release caused by hyperosmotic stimuli.

## Experimental procedures

### Protein purification, pull-down assays, and Western blots

The production of proteins in *Escherichia coli* and the preparation of cell lysates were performed as described (54, 55). *E. coli* cells were transformed with plasmids as listed in the resources table (Supporting information). Recombinant fusion proteins were purified by affinity chromatography using glutathione sepharose (GE Healthcare), amylase resin (NEB), or Ni–NTA agarose (Qiagen).

Pull-down assays were performed as described (55). Typically, 30 μl of glutathione sepharose affinity beads were pre-equilibrated and incubated with 12 μg of the purified protein of interest at 4 °C for 60 min in Tris-buffered saline buffer (150 mM NaCl, 50 mM Tris–HCl, and pH 7.5). The beads were washed three times with buffer, and potential binding proteins were incubated with the resins at 4 °C for 1 h. After washing, bound proteins were eluted with SDS sample buffer and analyzed by SDS-PAGE and Coomassie blue staining or visualized by immunoblotting using anti-GST antibodies (1:1000; rabbit, polyclonal, made inhouse).

The anti-TRPY1-4C9-A3 monoclonal rat serum antibody was generated inhouse (3, 13). Proteins of yeast cell lysates, prepared as described previously (56), were separated by SDS-PAGE and blotted onto polyvinylidene difluoride membranes (Thermo Fisher Scientific). Proteins were detected with horseradish peroxidase–coupled secondary antibodies (1:1000 dilution; Sigma–Aldrich) and the Western Lightning Chemiluminescence Reagent Plus (PerkinElmer) and the SuperSignal West Femto Maximum Sensitivity Substrate (Thermo Fisher Scientific). The antibody against Yrb1 was described (57).

### CaM binding

Purified MBP-fused proteins (12 μg) were immobilized to amylose resin and incubated with 12 μg of GST fusion proteins containing wt yeast CaM (Cmd1) or the non–Ca^2+^-binding yeast CaM mutant (Cmd1–6) in the presence (2 mM Ca^2+^) or the absence of Ca^2+^ (5 mM EGTA) for 60 min at 4 °C. After washing, bound proteins were eluted with SDS sample buffer, analyzed by SDS-PAGE blotted, and detected by anti-GST antibodies.

GST or GST-Cmd1 (12 μg) was immobilized to glutathione sepharose, and 6His-Sumo-TRPY1-N fusion proteins were added. The Sumo moiety was inserted to increase solubility. Alternatively, immobilized GST or GST-TRPY1-C was supplemented with 12 μg of 6His-Cmd1. After incubation with or without Ca^2+^ (2 mM CaCl_2_ or 5 mM EGTA) for 60 min at 4 °C, the resins were washed, and bound proteins were eluted with SDS and analyzed by SDS-PAGE and Coomassie blue staining.

### ^45^Ca^2+^ binding assay

The ^45^Ca^2+^ binding assay of blotted proteins was performed as described (58). The recombinant proteins were run on SDS-PAGE and blotted onto a nitrocellulose membrane, which was soaked for 120 min in buffer (60 mM KCl, 5 mM MgCl_2,_ 10 mM imidazole–HCl, and pH 6.8), changed every 30 min, followed by incubation in the same buffer containing 1 mCi/l ^45^Ca^2+^ for 10 min. Thereafter, the membrane was rinsed with deionized water, dried at 21 °C, and exposed to a phosphorimager screen (BAS-IP MS 2040; Fujifilm) for 12 to 24 h. The screen was scanned by a Typhoon FLA 9500 (GE Healthcare Life Sciences). The ^45^Ca binding to spotted peptides was essentially performed as described (13).

### Peptide scan assay and ^14^C-Cmd1 binding

About 20-mer peptides of the TRPY1 N and C terminus were synthesized on an Intavis ResPepSL peptide spot synthesizer and spotted onto hardened cellulose membranes at 16 nmoles per spot. The amino acid sequence was shifted by five amino acids from one spot to the next. The membranes were equilibrated with binding buffer (50 mM Tris, pH 7.5, 150 mM NaCl, and 0.1% Triton X-100) at room temperature for 60 min followed by incubation in the same buffer including 2 mM CaCl_2_ and 60 cpm of ^14^C-GST-Cmd1 per peptide spot at 4 °C with gentle shaking for 16 h. Unbound proteins were washed away using binding buffer, and membranes were air dried. Membranes were exposed to phosphorimager screens for 48 h, and screens were scanned by using the Typhoon FLA 9500 (GE Healthcare Life Sciences).

### Yeast strains and plasmids

The TRPY1 knockout yeast strain (*Δyvc1*) GSY1180 (*MATα YVC1::TRP1*) and the CaM mutant strain *cmd1–6* (*MATα cmd1–6*) (35) are isogenic to the wt strain W303 (GSY170, *MATα ura3 leu2 his3 trp1 ade2 can1*). The *cmd1–6* strain is a kind gift from Dr Trisha Davis, Seattle, WA. For Ca^2+^ imaging or patch clamp experiments, yeast cells were transformed with plasmids as listed in the resources table (Supporting information). Cells were cultured overnight in liquid yeast extract peptone dextrose or synthetic defined media (both Sigma–Aldrich) at 30 °C with rotary shaking and harvested at an absorbance at 600 nm between 1.2 and 1.5.

### Cytosolic Ca^2+^ measurements in yeast

wt, CaM-mutant *cmd1–6*, and yvc1-deficient (*Δyvc1*) yeast cells were transformed with plasmid *pEVP11-AEQ89* (9), encoding the photoprotein aequorin, to measure cytosolic-free Ca^2+^ concentrations. For some experiments, the yeast cells were cotransformed with the plasmid *pRS426-CMD1*, encoding the yeast CaM. Ca^2+^ imaging was carried out as described (13). In brief, cells were resuspended in fresh medium to a density of an absorbance of 10 at 600 nm. Coelenterazine (Synchem) was added at a final concentration of 60 μM. After incubation for 20 min at 30 °C, cells were pelleted and resuspended in fresh medium and incubated again for 45 to 90 min at 30 °C on a roller. Luminescence was detected at 30 °C at 470 nm using a microplate reader (Infinite M200; Tecan) and plotted as relative luminescence units over time using the i-control 1.7 microplate reader software (Tecan).

### Patch clamp experiments on yeast vacuoles

The preparation of large vacuoles was performed as described (2, 13). Briefly, overnight yeast cell cultures of wt, CaM-mutant *cmd1–6*, and yvc1-deficient (*Δyvc1*, for some experiments cotransformed with plasmids as listed in the resources table [Supporting information]) were harvested at an absorbance of 1 to 1.5 at 600 nm and kept in incubation buffer (50 mM KH_2_PO_4_, 0.2% β-mercaptoethanol, and pH 7.2) for 15 min at 30 °C. To remove cell walls, protoplasting buffer (50 mM KH_2_PO_4_, 0.2% β-mercaptoethanol, 2.4 M sorbitol, and pH 7.2) was added with final concentrations of 1 mg/ml zymolyase 20T (ICN Biochemicals) and 150 mg/ml (324 mM) bovine serum albumin (fraction V protease free; Carl Roth). After 45 min of incubation, spheroplasts were pelleted and resuspended in stabilizing buffer (220 mM KCl, 10 mM CaCl_2_, 5 mM MgCl_2_, 5 mM MES, 1% [w/v] glucose, and pH 7.2). After 2 to 3 days, the spheroplasts revealed giant vacuoles.

To release vacuoles, spheroplasts were incubated in releasing buffer (100 mM potassium citrate, 5 mM MgCl_2_, 10 mM glucose, 10 mM MES, and pH 6.8) for 2 to 5 min. Then releasing buffer was replaced by bath (cytosolic side) solution (150 mM KCl, 5 mM MgCl_2_, 2 mM DDT, 10 mM Hepes, and pH 7.2). A Zeiss Axiovert 135 microscope equipped with a 40× LD Achroplan objective (Zeiss) was used to visualize the vacuoles. Patch pipettes were pulled from glass capillaries GB150T-8P (Science Products) at a PC-10 micropipette puller (Narishige) and filled with the same solution as in the bath (see aforementioned), leading to resistances between 2 and 4 MΩ. After reaching the giga-seal, short voltage pulses (850–1100 mV, 1–5 ms) were applied to break-in and reach the whole-vacuole configuration. About 1 mM CaCl_2_ or BaCl_2_ was applied directly onto the measured vacuole *via* an application pipette (cytosolic side), or 1 mM CaCl_2_ was added into the patch pipette (vacuolar side). Currents were recorded from voltage ramps of 200 ms spanning from 150 to −150 mV from a holding potential of 0 mV applied every 2 s using an EPC-9 patch clamp amplifier (HEKA). Currents were normalized to the size (capacitance) of the vacuole and plotted as pA/pF (current densities). Inward and outward currents were extracted at −80 and 80 mV, respectively, and plotted *versus* time. Representative current–voltage relationships were extracted at indicated time points. Note that the membrane potentials refer to the cytosolic side, that is, inward currents at −80 mV represent movement of positive charges from the vacuole toward the cytosol (36).

### Cytosolic Ca^2+^ measurements in HEK-293 cells

For cell transfection, we used either the ponasterone A–inducible expression system (Invitrogen) or the bicistronic expression vector *pcAGGS-IRES-GFP* (13, 59). HEK-293 cells (American Type Culture Collection; CRL 1573) were cultured in Dulbecco's modified Eagle's medium (Thermo Fisher Scientific) with 10% fetal bovine serum and 1% penicillin/streptomycin. HEK-293 cells were plated on glass coverslips and after reaching about 80% confluency cotransfected with *pIND-TRPY1-IRES-GFP* (HEK TRPY1) or *pIND-IRES-GFP* (HEK control) plus pVgRXR (Invitrogen) plasmids. About 24 h after transfection, 10 μM ponasterone A (Invitrogen) was applied to induce *YVC1* expression. Fugene HD (Promega) was used as transfection reagent. Cells were used 24 to 72 h after transfection.

For Ca^2+^ imaging, cells were loaded in media with 5 μM Fura-2-acetoxymethylester (TEFLabs) for 30 min at 37 °C. Subsequently, cells were washed and placed into a bath chamber containing 140 mM NaCl, 2.8 mM KCl, 2 mM MgCl_2_, 10 mM Hepes, 10 mM glucose, and pH 7.2 in the presence or the absence of 1 mM CaCl_2_. Compounds were applied directly into the bath. The Ca^2+^-dependent Fura-2 fluorescence of single cells was monitored at a rate of 0.5 Hz with a dual excitation fluorometric imaging system (polychrome V; TILL Photonics) controlled by TILLvisION software (TILL Photonics), using a Zeiss Axiovert 200 M microscope equipped with a 20× Zeiss EC Plan Neofluar objective. Fura-2-loaded cells were excited at 340 and 380 nm for 30 ms every 2 s each, and the fluorescence emission above 450 nm was detected by an Andor iXon CCD camera. After background subtraction, positively transfected cells, identified by their GFP expression (*green fluorescence*), were marked as regions of interest, and their mean fluorescence at 340 nm (F_340_) and 380 nm (F_380_) excitation were computed into relative ratio units (F_340_/F_380_). To analyze hypertonic shock–mediated volume changes, cells were excited by the Fura-2 Ca^2+^-independent wavelength 360 nm for 30 ms every 2 s and the fluorescence emission above 450 nm was detected and normalized to the fluorescence intensity just before the hypertonic shock (F/F_120s_).

### Cellular localization of TRPY1

HEK-293 cells were transfected with cDNA for TRPY1 3′ extended by the cDNA of GFP (*pcDNA3-TRPY1-GFP*) to yield TRPY1-C-GFP fusion proteins (TRPY1-GFP), the ER marker ER-CFP (*pECFP-ER*; catalog no. 6907-1; Clontech) and/or the ER chaperon BiP, fused to mRFP (*pN1-BiP-mRFP-KDEL*, subcloned from *pN1-BiP-mGFP-KDEL*, addgene plasmid #62231 (60); BiP-mRFP). About 48 h after transfection, images were captured with a Plan-Neofluar 63×/1.25 Oil or Plan-Neofluar 100×/1.3 Oil objective (both ZEISS) at a fluorescence microscope (Observer Z1; ZEISS), equipped with GFP, ECFP, and Cy3 filter sets (all from AHF Analysentechnik) and a CCD AxioCam MRm camera (ZEISS), using AxioVision software (ZEISS). Images were cropped and merged using Fiji (ImageJ; National Institutes of Health).

### Patch clamp experiments on HEK-293 cells

HEK-293 cells were cultured in Dulbecco's modified Eagle's medium (Thermo Fisher Scientific) with 10% fetal bovine serum and 1% penicillin/streptomycin, plated on glass coverslips (2.5 cm in diameter) and, after reaching about 80% confluency, transfected with *pcAGGS-TRPY1-IRES-GFP*, and for some experiments, cotransfected with either *pcAGGS*-CaM-*IRES-GFP* or *pcAGGS*-CaM (*D*_*EF1,2,3,4*_*A*)*-IRES-GFP*. About 24 h after transfection, cells were trypsinized and scattered on small glass coverslips (1 cm in diameter). Patch clamp experiments were performed 48 to 72 h thereafter. A Zeiss Axiovert 135 microscope equipped with a 40× LD Achroplan objective (Zeiss), a *green* LED (Rapp OptoElectronic) and a GFP filter set (AHF Analysentechnik) were used to visualize and identify *green* cells. The bath solution contained 140 mM NaCl, 2.8 mM KCl, 3 mM MgCl_2_, 10 mM Hepes, and 10 mM glucose (pH 7.2, adjusted with NaOH). Patch pipettes were pulled from glass capillaries GB150T-8P (Science Products) at a PC-10 micropipette puller (Narishige) and filled with pipette solution comprising 120 mM cesium glutamate, 8 mM NaCl, 1 mM MgCl_2_, 10 mM Hepes, 10 mM Cs-BAPTA (pH 7.2, adjusted with CsOH), leading to resistances between 3 and 4 MΩ. For activation of TRPY1, the pipette solution either contained 1 μM free Ca^2+^ (10 mM Cs-BAPTA + 8.2 mM CaCl_2_) or bath solution, supplemented with 500 mM sorbitol (hypertonic shock), was applied *via* an application pipette directly onto the measured cell with 300 nM free Ca^2+^ (10 mM Cs-BAPTA + 5.7 mM CaCl_2_) in the patch pipette. Intracellular-free Ca^2+^ concentrations were calculated using webmaxc standard (https://somapp.ucdmc.ucdavis.edu/pharmacology/bers/maxchelator/webmaxc/webmaxcS.htm). For some experiments, 10 μM recombinant bovine (mammalian) CaM (Sigma–Aldrich) was added to the patch pipette, or Ca^2+^ was omitted (0Ca_i_). For tip coating, patch pipettes were dipped into sigmacote (Sigma–Aldrich). Whole-cell currents were recorded from voltage ramps of 400 ms spanning from −100 to 100 mV from a holding potential of 0 mV applied every 2 s using an EPC-9 patch clamp amplifier (HEKA). Currents were normalized to the size (capacitance) of the cell and plotted as pA/pF (current densities). Inward and outward currents were extracted at −80 and 80 mV, respectively, and plotted *versus* time. Representative current–voltage relationships were extracted at indicated time points.

### Analysis and statistics

The hydrophilicity plot was performed using Protean (DNASTAR). Luminometric Ca^2+^ imaging data were saved as Excel files. HEK-293 cells Ca^2+^ imaging data were initially analyzed in TILLvisION. Fluorescence images were captured by the AxioVision software (ZEISS) and processed in ImageJ. Patch clamp data were analyzed in Patchmaster or Fitmaster (HEKA). All data were finally transferred to IgorPro (WaveMetrics) for further analysis and graphical presentation. GraphPad Prism (GraphPad Software, Inc) was used to prepare bar graphs, scatter plots, and box plots as well as to test for statistical differences. All data were first tested for parametric or nonparametric distribution using Anderson–Darling, D'Agostino and Pearson, Shapiro–Wilk, and Kolmogorov–Smirnov tests. Means of parametric data were compared by Student's *t* test for two groups and one-way ANOVA with Bonferroni's multiple comparison post hoc test for more groups. Means from nonparametric data were compared by Mann–Whitney test for two groups and Kruskal–Wallis test with Dunn's multiple comparison post hoc test for more groups. The respective tests are indicated in the legends to the figures. Parametric data are presented as bar graphs, and nonparametric data as Tukey's box and whiskers with the boxes extend from the 25th to the 75th percentile (interquartile range), and the line inside the box shows the median. Whiskers are extended to the most extreme data point that is no more than 1.5× interquartile range from the edge of the box, and outliers beyond the whiskers are depicted as dots. Error bars represent the SEM or SD as indicated in the legends to the figures. Final figures were prepared in CorelDRAW (Corel Corporation).

## Data availability

All relevant data are contained within the article or the supporting information.

## Supporting information

This article contains supporting information (61, 62).

## Conflict of interest

The authors declare that they have no conflicts of interest with the contents of this article.

## Supporting information

Resource Table and Supplemental Figures S1–S4
