# Supplementary material for: Activity of the yeast vacuolar TRP channel TRPY1 is inhibited by Ca2+–calmodulin binding
Source: J Biol Chem. 2021 Aug 28;297(4):101126. doi: 10.1016/j.jbc.2021.101126 (PMC8449268; doi:10.1016/j.jbc.2021.101126)
Supplement: Resource Table and Supplemental Figures S1–S4 [file mmc1.docx]

**Activity of the yeast vacuolar TRP channel TRPY1 is inhibited by Ca^2+^-calmodulin binding**

Mahnaz Amini^1,2,§^, Yiming Chang^1,2,3,§^, Ulrich Wissenbach^1^, Veit Flockerzi^1^, Gabriel Schlenstedt^2^ and Andreas Beck^1*^

^1^ Experimentelle und Klinische Pharmakologie und Toxikologie / PZMS, Universität des Saarlandes, 66421 Homburg, Deutschland

^2^ Department of Medical Biochemistry and Molecular Biology / PZMS, Medical School, Saarland University, 66421 Homburg, Germany

^3^ present address, Evolva Group, 4153 Kanton Reinach, Switzerland

^§^ these authors contributed equally to this study

**Supporting Information**

**Resources table**

| REAGENT or RESOURCE | SOURCE | IDENTIFIER |
| --- | --- | --- |
| Antibodies | | |
| rat monoclonal anti-TRPY1 | in-house (3, 13) | N/A |
| rabbit polyclonal anti-GST | Sigma Aldrich | G7781 |
| rabbit polyclonal anti Yrb1 | (57) | N/A |
| rabbit polyclonal anti-calnexin | Stressgen Biotech. Corp. | SPA-865 |
| mouse monoclonal anti-MBP | Santa Cruz Biotech. | Sc-13564 |
| goat anti-rat-IgG-horseradish peroxidase-linked | Amersham Biosciences | NA935 |
| donkey anti-rabbit-IgG peroxidase-linked | Amersham Biosciences | NA9340 |
| Bacterial and Virus Strains | | |
| *E. coli* Rosetta (DE3)-pLysS | Novagen | 70956 |
| Chemicals, Peptides, and Recombinant Proteins | | |
| recombinant TRPY1 fragments | this study | N/A |
| Fugene HD | Promega | E2311 |
| FURA-2 AM | TEFLabs | 0103 |
| Zymolyase 20T | ICN Pharmaceuticals |  |
| Ophiobolin A | Cayman Chemical | Cay15381 |
| Coelenterazine | Synchem | s053 |
| recombinant bovine (mammalian) calmodulin | Sigma-Aldrich | C4874 |
| BAPTA tetracesium salt (Cs-BAPTA) | Biotium | 50001 |
| Sigmacote | Sigma-Aldrich | SL2 |
| Experimental Models: Cell Lines | | |
| HEK-293 | ATCC | CRL1573 |
| Experimental Models: Organisms/Strains | | |
| S. cerevisiae wild-type strain W303 (GSY170 [MATα ura3 leu2 his3 trp1 ade2 can1])* | (3, 13) |  |
| Δyvc1 (GSY1180 [MATα YVC1::TRP1]) | (3) |  |
| Cmd1-6 [MATα cmd1-6] | (35) |  |
| Recombinant DNA | | |
| *Procaryotic expression plasmids (E. coli)* | | |
| pGEX-4T | GE Healthcare | 28-9545-49 |
| pGEX-4TEV | (54) | N/A |
| pGST-Cmd1 | this study | N/A |
| pGST-Cmd1-6 | this study | N/A |
| pGST-TRPY1-N | this study | N/A |
| pGST-TRPY1-C | this study | N/A |
| pMal | New England BioLabs | E8200 |
| pMal-TRPY1-N | this study | N/A |
| pMal-TRPY1-C | this study | N/A |
| pET-14b (6His) | Novagen | 69660-3 |
| pET-14b-cmd1 | this study | N/A |
| pCA528 (His6-SUMO) | (61) | N/A |
| pCA528-TRPY1-N | this study | N/A |
| *Expression plasmids for HEK-293 cells* | | |
| pIND and pVgRXR (ponasterone A-inducible expression in mammalian cells) | Invitrogen Ltd, Paisley, UK | K1001-01, K1003-01, K1004-01 |
| pIND-IRES-GFP | this study | N/A |
| pIND-TRPY1-IRES-GFP | this study | N/A |
| pcAGGS-IRES-GFP | (59) | N/A |
| pcAGGS-TRPY1-IRES-GFP | this study | N/A |
| pcAGGS-calmodulin-IRES-GFP | this study | N/A |
| pcAGGS-calmodulin(D_EF1,2,3,4_A)-IRES-GFP | this study | N/A |
| pcDNA3-TRPY1-GFP (TRPY1-GFP) | this study | N/A |
| pECFP-ER (ER-CFP) | CLONTECH, Heidelberg, Germany | Cat. #6907-1 |
| pN1-BiP-mRFP-KDEL (BiP-mRFP) | this study, subcloned from pN1-BiP-mGFP-KDEL, addgene plasmid #62231 (60) | N/A |
| *Expression plasmids for yeast* | | |
| pEVP11-AEQ89 | (8) | N/A |
| pRS316 | (62) | N/A |
| pRS426-CMD1 | this study | N/A |
| pRS316-YVC1 | this study | N/A |
| pRS316-YVC1-K86 89 91A | this study | N/A |
| pRS316-YVC1-K43 48K | this study | N/A |
| pRS316-YVC1-Δ33-92 | this study | N/A |
| Software and Algorithms | | |
| TILLvisION | TILL Photonics | N/A |
| IGOR Pro | WaveMetrics | https://www.wavemetrics.com/products/igorpro/igorpro.htm |
| Patchmaster / Fitmaster | HEKA Elektronik GmbH | https://www.heka.com/downloads/downloads_main.html#down_patchmaster_next |
| Prism | GraphPad | https://www.graphpad.com/scientific-software/prism/ |
| DNASTAR | DNASTAR | https://www.dnastar.com/?gclid=EAIaIQobChMIys_a8Yes6QIVBNZ3Ch2OuAPHEAAYASAAEgL9z_D_BwE |
| CorelDRAW | Corel Corporation | https://www.coreldraw.com/de/ |
| AxioVision | ZEISS | https://www.micro-shop.zeiss.com/de/de/system/software-axiovision+software-produkte/1007/ |
| Other | | |
| micropipette puller | Narishige | PC-10 |
| patch clamp amplifier | HEKA Elektronik GmbH | EPC-9 |
| glass capillaries | Science Products | GB150T-8P |
| biomolecular imager | GE Healthcare | Typhoon FLA 9500 |
| microplate reader | Tecan | Infinite M200 |
| microscope patch clamp | ZEISS | Axiovert 135 |
| microscope Ca^2+^ imaging | ZEISS | Axiovert 200 |
| microscope fluorescence images | ZEISS | Observer Z1 |
| monochromator | TILL Photonics | Polychrome 5 |
| CCD camera Ca^2+^ imaging | Andor | iXon |
| CCD camera fluorescence images | ZEISS | AxioCam MRm |
| Green LED light source | Rapp OptoElectronic | KSL 70 (470 nm) |
| air pressure driven bath application system | Lorenz Messgerätebau | MPCU3 |
| micromanipulator | Sensapex | uMp |

* The *yvc1* knockout yeast strain GSY1180 (*MATα YVC1::TRP1*) and the calmodulin mutant strain *cmd1-6* (*MATα cmd1-6*) (34) are isogenic to the wild-type strain W303 (GSY170, *MATα ura3 leu2 his3 trp1 ade2 can1*). The cDNAs of mammalian calmodulin (NCBI accession number NP_001734), yeast calmodulin (accession number KQC45519.1), and TRPY1 (accession number NM_001183506.1) were cloned and subcloned in the laboratory of the authors (this study and (3, 13, 23)).

(For references see main text.)

**Figure S1:** Ba^2+^ activates TRPY1 currents in the wild-type and in the cmd-1-6 mutant yeast strain, and an outward conductance in the TRPY1 knockout strain Δyvc1

**

In- and outward currents at -80 and 80 mV, extracted from 200 ms voltage ramps (0.5 Hz) spanning from 150 to -150 mV, V_h_ 0 mV, plotted versus time, measured in vacuoles from wild-type (wt) and *cmd1-6* mutant yeast strains (A) and from the *TRPY1*-deficient (*Δyvc1*) yeast strain (D). The bars in A and D indicate cytosolic application of 1 mM Ba^2+^. (B, D) Corresponding IVs (B) and inward current amplitudes (C) of the net Ba^2+^-mediated TRPY1 current (I_max_ (B), I_plateau_ (C)) in A, with current amplitudes right before application of 1 mM Ba^2+^ subtracted. Numbers of measured vacuoles are indicated in brackets. Data represent means (B), means ± S.E.M (A, D) and means ± S.D. (C) with p value in C calculated by an unpaired two-tailed student’s t-test.

**Figure S2:** Functional plasma membrane localization of GFP-fused TRPY1

In- and outward currents at -80 and 80 mV, extracted from 400 ms voltage ramps (0.5 Hz) spanning from -100 to 100 mV, V_h_ 0 mV, plotted versus time, measured in HEK-293 cells transfected with cDNA for TRPY1 3` extended by the cDNA of GFP to yield TRPY1-C-GFP fusion proteins (TRPY1-GFP; A) or non-transfected HEK-293 cells (B) challenged by 1 µM (A, B) or 100 nM (A, grey traces) intracellular Ca^2+^ infused via the patch pipette. Corresponding current-voltage relations (IVs) of the currents at 200 s (I_200 s_) are shown in the right panels. The numbers in brackets indicate the number of measured cells.

**Figure S3:** TRPY1 currents in HEK-293 cells are reduced by intracellular CaM

In- and outward currents at -80 and 80 mV, extracted from 400 ms voltage ramps (0.5 Hz) spanning from -100 to 100 mV, V_h_ 0 mV, plotted versus time, measured in HEK-293 cells transfected with TRPY1 cDNA (A, D, H, N) or in non-transfected HEK-293 cells (L) challenged by 1 µM intracellular Ca^2+^ (A) or hyperosmotic shock (500 mM sorbitol; D, H, L, N) in the absence (N; 10 mM BAPTA, 0Ca_i_) and in the presence of 300 nM Ca^2+^ in the patch pipette (D, H, L). (A, D) Pipette solution without (black) or with 10 µM CaM (red). In H HEK-293 cells were co-transfected with mammalian calmodulin (CaM; red), the non-Ca^2+^-binding mammalian calmodulin mutant (CaM(D_EF1,2,3,4_A); blue) or empty vector as control (black). B, E, F, I, J, M, O show the corresponding current-voltage relations (IVs) of the currents in A, D, H, L and N at 240 s (I_240 s_; B, F, J, M, O) or 200 s (I_200 s_, E, I). The numbers in brackets indicate the number of measured cells. (C, G, K) Summary of the current amplitudes at -80 mV extracted at 240 s (C, G and K, right) and 200 s (G and K, left) from the experiments in A, D and H. Data in B, E, F, I, J, M, O represent means, data in A, D, H, L and N means ± S.E.M.. Data in C, G and K are presented as Tukey`s box and whiskers with the boxes extend from the 25th to the 75th percentile (inter-quartile range, IQR), and the line inside the box represents the median. Whiskers are extended to the most extreme data point that is no more than 1.5×IQR from the edge of the box and outliers beyond the whiskers are depicted as dots. The indicated p values were calculated by Mann-Whitney test (C, G) and Kruskal-Wallis test (K, left, p=0.4458; K, right, p=0.6637) with Dunn’s multiple comparison test.

**Figure S4:** Peptide scan to identify the binding sites on TRPY1 for yeast calmodulin (cmd1)

﻿Autoradiographs obtained after incubation of spotted peptides (approximately 16 nmoles per spot) in the presence of ^14^C-labeled GST-Cmd1. Peptides corresponding to the N- (A) and C-terminus (B) of TRPY1, with a length of 20 amino acids each, were synthesized on a cellulose membrane and the amino acid sequences were shifted by 5 amino acids from one spot to the other. Numbers on the left indicate the position of the starting amino acid residue in the TRPY1 primary structure.
